# Editorial: Revisiting the thymus: the origin of T cells

**DOI:** 10.3389/fimmu.2023.1197066

**Published:** 2023-04-13

**Authors:** Xiaoxi Lin, Yayi Gao, Yi Ding, Bin Zhao

**Affiliations:** ^1^ National Clinical Research Center for Metabolic Diseases, Metabolic Syndrome Research Center, Key Laboratory of Diabetes Immunology, Ministry of Education, and Department of Metabolism and Endocrinology, The Second Xiangya Hospital of Central South University, Changsha, Hunan, China; ^2^ Laboratory of Immune Cell Biology, Center for Cancer Research, National Cancer Institute, National Institutes of Health, Bethesda, MD, United States; ^3^ T Cell Biology and Development Unit, Laboratory of Genome Integrity, Center for Cancer Research, National Cancer Institute, National Institutes of Health, Bethesda, MD, United States; ^4^ Furong Laboratory, Central South University, Changsha, China

**Keywords:** T cell development, thymus, thymocyte, T cell differentiation, T cell signaling

T cells are crucial components of adaptive immunity, playing an indispensable role in protecting against external pathogenic agents and maintaining immunological homeostasis. T cell development in the thymus involves a series of highly structured procedures, including lineage commitment, T cell receptor (TCR) rearrangement, positive and negative selection, and other checkpoints (1). Disruption in this process can lead to T cell dysfunction, potentially leading to severe immunodeficiency or autoimmune diseases. Numerous studies have demonstrated that T cell development is a complex process, regulated by multiple factors and intricate signaling pathway networks (1, 2). Despite significant advancements in this field, unraveling the underlying mechanisms for T cell development remains a formidable challenge. Therefore, the purpose of this Research Topic was to collect evidence for a deeper understanding of underlying mechanisms involved in thymocyte development as this will not only expand our knowledge of cell fate determination through gene regulatory networks but also could pave the way for potential immunotherapeutic strategies.

The thymus provides a microenvironment to support T cell development, including stromal cells, primarily thymic epithelial cells (TECs), and other immune cells. Although TECs have been extensively studied for their role in T cell selection, the contribution of other immune cells such as macrophages and dendritic cells (DCs), to T cell development remains to be elucidated. Wang et al. provided a systemic review of the heterogeneity of DC and macrophage subpopulations in the thymus and their potential roles in T cell selection and maintenance of thymic homeostasis. Original research by Hou and Yuki showed that CD11c, a surface marker of DCs, is important for maintaining T cell survival as *Itgax* (encodes CD11c) deficiency leads to increased apoptosis of DP, CD4SP, and CD8DP T cells. These findings suggest that thymic DCs may contribute to supporting T cell survival through CD11c.

T cell development is regulated by multiple levels, including gene mutation, transcriptional regulation, epigenetic modification, and post-translational modifications. Gene mutations can affect T cell development, especially some key mutations that can lead to severe combined immunodeficiency (SCID). Sertori et al. explored a causal etiology in a patient with T-B+NK+ immunodeficiency and found the MED14^V763A^ variant that may be responsible for the disease. However, this deficiency can be observed in neither male siblings of the proband nor mouse models carrying the same variant. Therefore, one possibility is that the MED14^V763A^ variant may contribute to the disease, but the degree and direction of this effect may be modulated by specific modifier genes that interact with MED14 to enhance or suppress its expression or function. Another possibility is that the disease may result from another unknown noncoding mutation. Transcription factors play a critical role in T cell development by regulating the expression of target genes involved in cell differentiation, proliferation, survival, and maturation. Bao et al. provided a comprehensive review of key transcription factors and epigenetic regulatory factors in each stage of T cell development, including both classic transcription factors such as T-cell-specific transcription factor (TCF-1) and Runx transcription factor family, and some newly reported transcription factors such as Zinc finger protein Yin Yang 1 (YY1). However, the roles of newly discovered transcription factors are less clear. Ji et al. demonstrated the importance of serine/arginine-rich splicing factor 1 (SRSF1) in T cell development by regulating the expression of the key transcription factor Runx3. Specifically, they show that the selective knockout of SRSF1 in the DP stage (SRSF1^fl/fl^CD4^cre^) results in decreased CD8SP cell numbers and maturation defects, which can be partially rescued by the overexpression of Runx3. However, the exact mechanism by which SPSF1 regulates the expression of Runx3 requires further investigation. In addition, as an important form of post-translational modification, protein ubiquitination regulates various biological processes, including T cell development. Zhong et al. reviewed the molecular mechanisms and cellular pathways that regulate thymocyte ubiquitination and focused on the roles of E3 ligases and deubiquitinating enzymes (DUBs).

Previous studies primarily focus on transcriptional regulation and post-translational modification. However, recent research highlights the importance of cell metabolism and metabolic reprogramming during T cell development in adapting to specific functional requirements. Zhang et al. provided an overview of the metabolic changes that occur during T cell development and summarized key metabolic regulators and regulation mechanisms. Furthermore, mitochondrial oxidative phosphorylation (OXPHOS) is central to cell metabolism and regulates various critical cellular processes, including proliferation, apoptosis, and differentiation by supplying energy and metabolites. The original research from Limper et al. reported that maintaining a high-fidelity replication of mtDNA is crucial for T cell development. They found that accumulated mtDNA mutations can impede proliferation during the DN stage and decrease mitochondrial density. This may be attributed to the dependence on mitochondria to provide energy and substrates during the highly proliferative DN3 stage and mitochondrial function is impaired by accumulated mtDNA mutations.

The interaction between major histocompatibility complex (MHC) and TCR is a critical link in T cell development. TCR signaling strength contributes to the determination of the fate of thymocytes. The E protein is one of the key transcription factors involved in T cell development and is negatively regulated by the Id protein, but the specific regulatory mechanism remains unclear. Anderson proposed the “Clutch” model to describe this process and discussed how Id3 participates in the development of T cells by regulating the activity of the E protein. They proposed that Id3 changes the accessibility of E protein target genes and guides T cells into different developmental pathways. During T cell development, the activity of Id3 is mainly regulated by the strength of the TCR signal. For example, pre-TCR transmits weaker TCR signals, which reduces the activity of Id3 and promotes the development of αβT cells. In contrast, γδ-TCR transmits stronger TCR signals and guides thymocytes to develop into γδT cells. Besides, CD4/CD8 lineage fate determination is also regulated by TCR signals. Stronger TCR signaling favors CD4 T cell development, whereas weaker TCR signaling favors CD8 T cell development.

It is widely accepted that MHC restriction is the key to T cell development, leading to the selection of functional and self-tolerant T cells. However, recent studies have shown that MHC restriction may not always be necessary for αβT cell development. Van Laethem et al. revealed the mechanism of MHC-independent αβTCR selection and the potential non-MHC ligands that may be involved in this process. DP thymocytes require TCR signals to maintain their survival after TCR rearrangement, but the initiation of αβTCR signaling relies on Lck tyrosine kinase, which binds to the coreceptors CD4 and CD8. Lck is typically sequestered by the coreceptors and TCRs can recruit Lck and initiate downstream signaling only when they bind to MHC-antigen peptide complexes corresponding to the coreceptors. However, without coreceptors sequestered, TCRs can recognize non-MHC ligands, such as CD155, CD102, and CD48, and complete signal transduction through free Lck to guide the development and maturation of MHC-independent αβT cells. However, it is worth noting that free Lck is not capable of transmitting high-affinity TCR signals effectively; therefore, it may not effectively clear self-reactive T cells.

In addition to the factors discussed earlier, sex steroids are found to play a role in T cell development as well. However, the direct effects of sex steroids on thymocytes and underlying mechanisms remain unclear. Taves and Ashwell concluded on the expression of sex steroid receptors on thymic cells and TECs and the mechanisms by which sex steroids regulate T cell development. In this review, they emphasized the suggestive finding of sex steroid production within the thymus itself.

The thymus not only contributes to central immune tolerance but also plays a critical role in establishing immune tolerance by generating immunosuppressive T cell subsets that migrate to the periphery. Regulatory T cells (Tregs) are a critical subset of these T cells that play a crucial role in maintaining immune homeostasis by suppressing effector T cells. While Tregs can differentiate from CD4+ T cells in the periphery, most Tregs are generated directly in the thymus, but their development is not completely understood. While TCR/CD28 co-stimulation and cytokine IL-2 are thought to be involved, a recent review by Tang et al. discussed the role of cytokines in thymic Treg (tTreg) development. The development of TCRαβ+CD8αα+ and TCRγδ+CD8αα+ intestinal intraepithelial lymphocytes (IELs), which play a role in the intestinal immune barrier and immune regulation, also originates in the thymus. Gui et al. concluded that the pivotal molecules and their functions are involved in the development process of these two specific IEL subsets.

In summary, these findings underscore the intricate and dynamic nature of T cell development, emphasizing the importance of a comprehensive understanding of the mechanisms governing this process.

## Author contributions

XL wrote the first draft of the present Editorial article. YG, YD and BZ revised it and provided their valuable and precious comments and suggestions. All authors contributed to the article and approved the submitted version.
